# Case Report: Severe ophiasis-pattern alopecia areata with concomitant atopic dermatitis in a 5-year-old boy successfully treated with dupilumab

**DOI:** 10.3389/fped.2025.1517769

**Published:** 2025-02-10

**Authors:** Zhendong Wei, Chao Yu, Aoxue Wang

**Affiliations:** Department of Dermatology, The Second Hospital of Dalian Medical University, Dalian, China

**Keywords:** alopecia areata, ophiasis-pattern alopecia areata, atopic dermatitis, pediatric patients, Th2 signaling, dupilumab

## Abstract

Alopecia areata (AA) is the third most common reason for dermatological consultations among children. Despite the availability of numerous therapies for AA, including topical and systemic modalities, management options for children diagnosed with severe AA are limited due to the lack of safe and effective treatments suitable for long-term use. Herein, a case involving a 5-year-old boy with severe ophiasis-pattern AA and moderate atopic dermatitis (AD), who was successfully treated with dupilumab, is reported.

## Introduction

Alopecia areata (AA) is a common form of non-scarring alopecia characterized by acute hair loss in the absence of cutaneous inflammatory signs. AA affects up to 2% of the global population (1). Children are affected at higher rates, with a peak prevalence at 6 years of age (2). Although no significantly unpleasant symptoms are usually reported, AA can affect the quality of life of these patients and may lead to emotional disorders including depression and anxiety (3). The clinical manifestations of AA can vary from small, well-circumscribed round or oval alopecic patches to severe subtypes such as alopecia totalis (AT), alopecia universalis (AU), or ophiasis-pattern AA (4). Ophiasis-pattern AA is a rare subtype of AA that presents as symmetric band-like hair loss, typically involving the occipital and temporal regions. Ophiasis-pattern AA is notorious for its tendency to affect children and adolescents, and for its resistance to treatment (5). In clinical practice, the severity of AA is most commonly assessed using the Severity of Alopecia Tool (SALT) (6). A SALT score ≥20 indicates moderate to severe AA, warranting a general medical indication for systemic therapy (7, 8).

Currently, the only 2 systemic medications approved for AA by the European Medicines Agency (EMA) and Food and Drug Administration (FDA) are baricitinib [a Janus kinase (JAK) 1/2 inhibitor, for use in adults ≥18 years of age] and ritlecitinib (a JAK 3/TEC inhibitor, for use in individuals ≥12 years of age) (6). Overall, management options for moderate-to-severe AA in younger children are limited. The long-term use of off-label oral corticosteroids and other immunosuppressants is associated with many side effects (9). Dupilumab, a monoclonal antibody, has been approved by the EMA and FDA for the treatment of atopic dermatitis (AD) in patients ≥6 months of age. Recent evidence suggests that dupilumab promotes hair regrowth in patients with AA (10). However, information regarding its efficacy and safety in treating severe ophiasis-pattern AA, especially in children ≤6 years of age, is scarce. The present report describes a case of severe ophiasis-pattern AA (SALT score, 80) and moderate AD involving a 5-year-old boy who was successfully treated with dupilumab.

## Case presentation

A 5-year-old boy presented to our clinic with severe hair loss that persisted and worsened over the past 2 years. Previous treatments, consisting of the oral compound glycyrrhizin (75 mg/day) and topical clobetasol propionate 0.05% cream (once per day), administered over the previous 6 months, yielded no improvement. A three-year history of untreated generalized dry skin with recurrent pruritic erythema and plaques on the extremities (onset before 2 years of age) was reported. No other atopic disorders or first-degree relatives reported atopic diseases.

**Figure 1:**
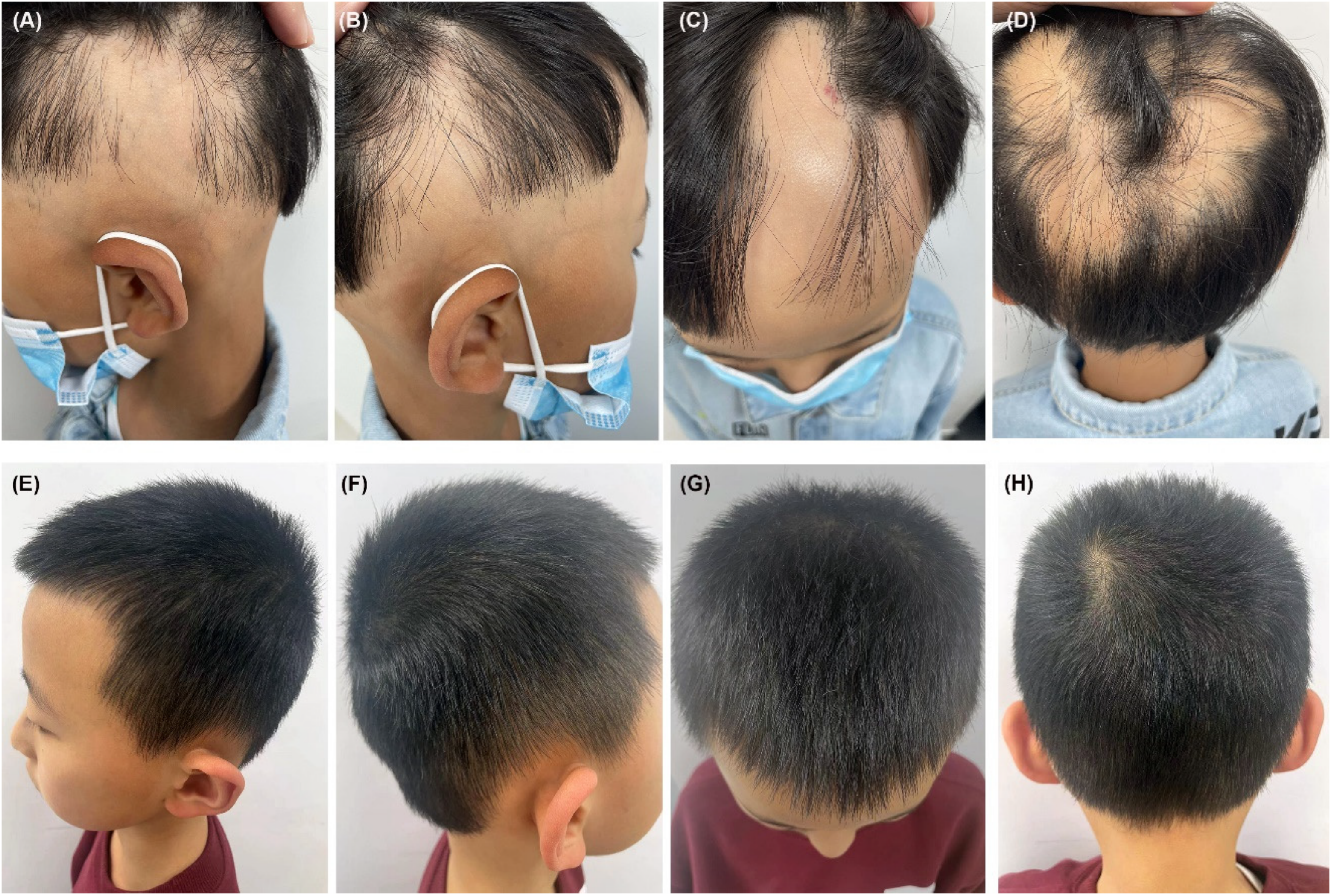
**(A–D)** Pretreatment, the patient demonstrated a band-like nonscarring alopecia affecting the posterior occipital, bilateral temporal, frontal and even parietal areas, with a SALT score of 80. **(E–H)** Six months after restarting dupilumab treatment, the patient achieved a complete regrowth of his scalp hair with his SALT score decreasing to 0.

**Figure 2:**
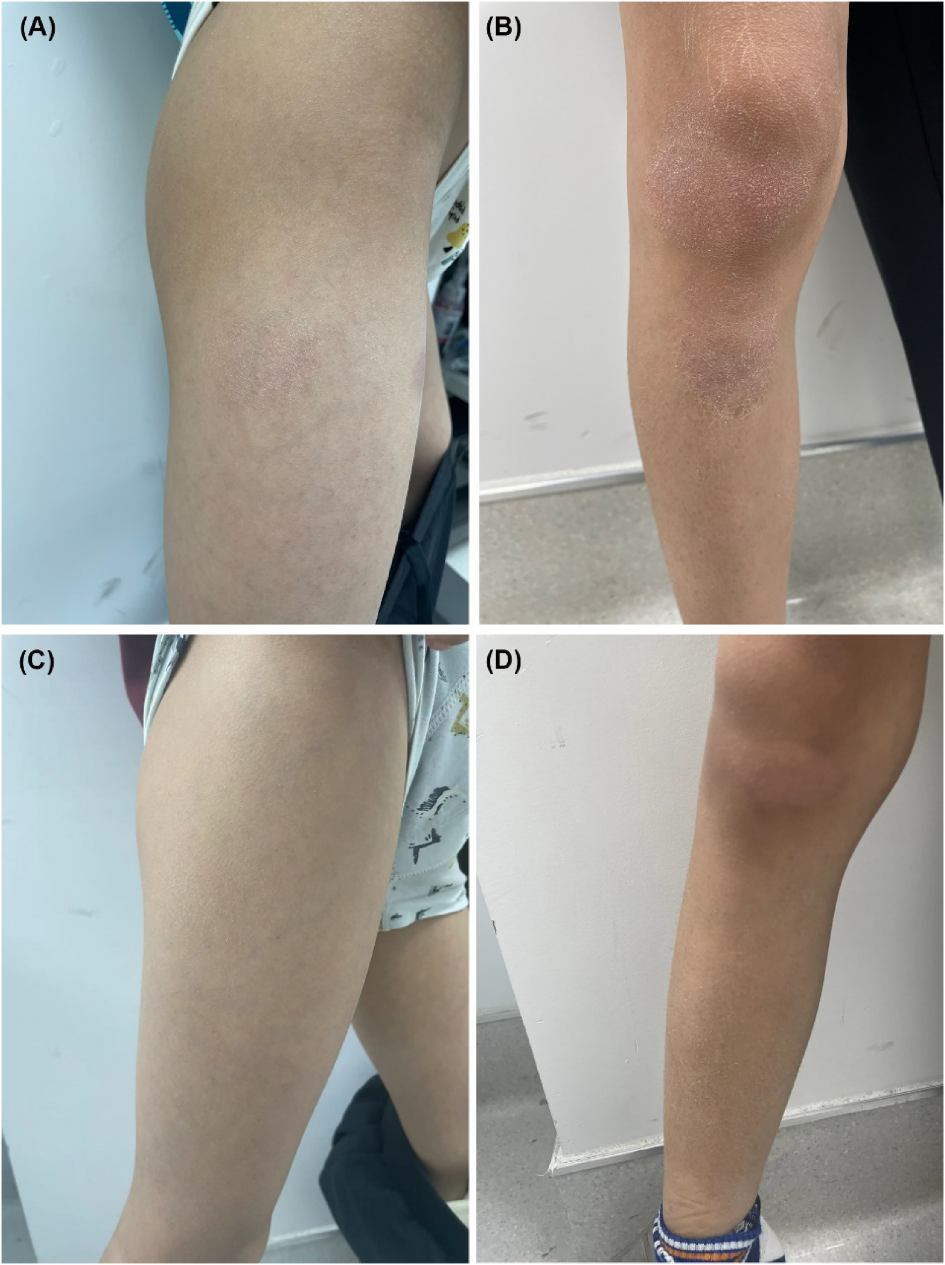
**(A,B)** Pretreatment, lichenified scaly plaques were scattered on the extremities, with a SCORAD score of 34. **(C,D)** Six months after restarting dupilumab treatment, significant improvements in the AD lesions were observed, with a SCORAD score of 8.

Physical examination revealed band-like nonscarring alopecia involving the posterior occipital, bilateral temporal, frontal and even parietal areas, with a SALT score of 80 (Figures 1A–D). Dermoscopy revealed exclamation marks, black dots, and broken hair (Supplementary Figure S1). No involvement of the eyebrows or nails was observed. In addition, pronounced xerosis was widespread throughout the body, with lichenified scaly plaques scattered symmetrically on the buttocks and extremities (Figures 2A,B), including the antecubital and popliteal fossae (flexural eczema). These symptoms fulfilled the Williams criteria for AD. The SCORing Atopic Dermatitis (SCORAD) score was 34 and the peak pruritus-numerical rating scale (PP-NRS) score was 8. Laboratory investigations revealed a serum immunoglobulin (Ig)E level of 184 IU/ml (normal range, 0–100 IU/ml). The differential blood count was within the normal range without eosinophilia. The patient was diagnosed with severe ophiasis-pattern AA and moderate AD.

Considering the safety requirements for use in young children and, after obtaining informed consent, dupilumab was prescribed as a 600 mg subcutaneous injection and maintained at 300 mg every 4 weeks thereafter, along with topical moisturizers. Although significant improvements in AD pruritus and lesions were observed after 2 weeks, no hair regrowth was achieved by the end of the third month of treatment. Dupilumab was discontinued at this time as requested by the patient's mother.

**Figure 3:**
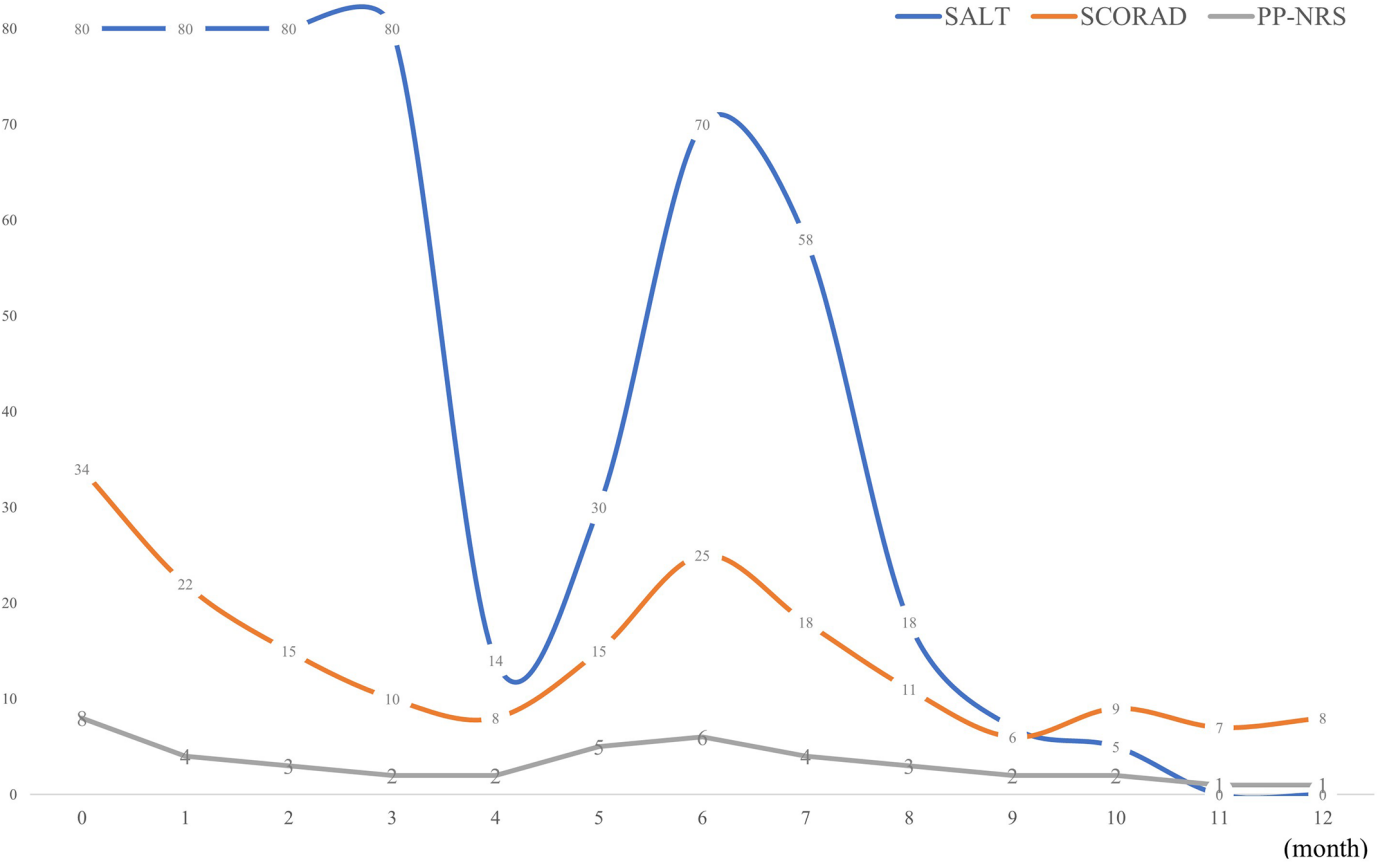
Summary of changes in SALT, SCORAD and PP-NRS scores over a one-year period. SALT, severity of Alopecia Tool; SCORAD, SCORing Atopic Dermatitis; PP-NRS, peak pruritus-numerical rating scale.

Unexpectedly, robust hair regrowth emerged during the fourth month, covering virtually the patient's entire scalp. However, this remarkable effect lasted <1 month, followed by deteriorating hair loss, which returned to pre-treatment, baseline levels. Dupilumab was restarted at the same dose and frequency as that used in the initial treatment. At the six-month follow-up after restarting dupilumab treatment, the patient's hair regrowth was complete. The SALT score decreased to 0 (Figures 1E–H), the SCORAD score was 8, and the PP-NRS score was 1 (Figures 2C,D). No adverse events were observed. A summary of the changes in the SALT, SCORAD, and PP-NRS scores during the one-year period of dupilumab treatment period is reported in Figure 3.

The patient was maintained on regular administration of dupilumab, and when assessed during follow-up in the second year, no recurrences of AA or AD were observed. The patient was monitored at regular follow-up appointments.

## Discussion

AA is one of the most common autoimmune diseases and the third most common reason for dermatological consultations among children (11). However, the lack of comprehensive understanding of the pathogenesis of this condition has severely hindered the development of specific therapies. Traditionally, AA is considered to be as T-helper (Th) 1 cell-related inflammatory disease. Activated CD8+NKG2D+T cells produce the Th1 cytokine interferon-gamma, leading to a disruption of immune privilege of hair follicles and the exposure of self-antigens. As a result, these hair follicles are vulnerable to autoimmune assault and hair loss (12).

The JAK/STAT signaling pathway is implicated in the loss of hair follicle immune privilege by interferon-gamma, suggesting that JAK inhibitors may provide a means for treating AA (13). Although 2 systemic JAK inhibitors have been approved for the treatment of AA, baricitinib for adults, and ritlecitinib for those ≥12 years of age, they are not fully effective in all cases. For adults with severe AA (SALT score ≥50), ≤41% achieved a SALT score ≤20 at week 52 in response to a daily administration of 4 mg baricitinib (14). In adolescents with severe AA, 25%–50% achieved a SALT score ≤20 at week 48 following a daily administration of 30–50 mg ritlecitinib (15). There are limited data regarding the efficacy of JAK inhibitors in the treatment of children with AA, with only single-patient case reports and a small case series available (11). Moreover, once treatments with JAK inhibitors are discontinued, hair shedding often resumes, with virtually all hair regrowth lost within approximately 3 months (9). Accordingly, treatment of AA with JAK inhibitors requires long-term use; however, safety data remain limited (10). Especially for those <12 years of age, treatment with off-label JAK inhibitors may pose additional safety concerns. Reported complications associated with JAK inhibitors include bacterial, fungal, mycobacterial and viral infections, along with lipid abnormalities, high liver enzyme levels, and leukopenia. Notably, baricitinib, ritlecitinib, tofacitinib, and upadacitinib have all received “black-box” warning labels regarding their increased risk for serious cardiovascular-related events such as heart attacks, stroke, and blood clots, and the potential for cancer and death (16). Thus, regular laboratory monitoring is necessary for all patients receiving oral JAK inhibitors. These findings highlight the need for the development of safer and more effective treatments for AA, particularly for patients <12 years of age.

Recently, the contributions of atopic background and Th2 immune axis to the pathogenesis of AA have received increased attention and emphasis. The interest in these factors is mainly based on the following: large-scale population studies reporting strong associations between atopic diseases and AA (1, 17); significant up-regulation of Th2-related immune products [i.e., interleukin [IL]-13, C-C motif chemokine ligand [CCL] 17, CCL18, CCL22, and CCL26] within the scalps of patients with AA (9, 18); and high levels of serum IgE, even in the absence of an atopic background, often observed in patients with AA (9, 19).

Dupilumab is a fully human monoclonal antibody that inhibits Th2 signaling by blocking IL-4Rα, a common subunit of IL-4 and IL-13 receptors. In 2022, dupilumab received FDA approval for the treatment of moderate-to-severe AD in patients >6 months of age (10, 20). In 2018, Penzi et al. (21) reported the first known case of full hair regrowth in a 13-year-old girl with AT and severe AD after 11 months of treatment with dupilumab. Subsequently, an increasing number of studies have described an improvement of AA in patients treated with dupilumab (22, 23). Cai et al. (24) reported a 4-year-old patient diagnosed with AU who was resistant to baricitinib but was successfully treated with dupilumab. To the best of our knowledge, this is the youngest age among the patients with AA who have been treated with dupilumab. Recently, results from a phase 2a study, involving 60 patients with AA (≥18 years of age), substantiated the efficacy and safety of dupilumab for use in adult cases of AA. Patients responding to this treatment were more likely to have an atopic background and/or elevated baseline IgE levels (10). In a subsequent scalp biopsy substudy, dupilumab significantly suppressed multiple Th2-related markers, coupled with a significant upregulation of hair keratins (1). In a more recent single-center observational study conducted in 2024, involving 14 children with both AA and AD receiving dupilumab, 86%, 71%, and 57% of these children achieved improvements in SALT scores of 50%, 75%, and 90%, respectively, after 48 weeks of treatment. Baseline IgE levels were positively correlated with improvements in SALT scores. In that cohort, dupilumab was well tolerated by all patients (9). To date, only 2 adult patients (33 and 34 years of age) with ophiasis-pattern AA have been treated with dupilumab. Both have experienced satisfactory curative effects (25, 26). Accordingly, the 5-year-old patient described in the present study represents the youngest patient with ophiasis-pattern AA who was cured by dupilumab. Overall, dupilumab has been established as a new and promising drug for the treatment of AA in both adults and children.

Conversely, results from several studies have indicated the novel development of AA in patients with AD receiving dupilumab (27, 28). Dupilumab treatment in Th1-dominant patients has been hypothesized to potentiate Th1 responses by blocking Th2 signaling, thereby inducing or exacerbating AA (20). Accordingly, effective treatment of AA requires individualized protocols. For example, patients with early-onset AD, atopic comorbidities, and high IgE levels play a predominant role in Th2 skewing and are more suitable candidates for dupilumab treatment (9, 10). As observed in the current case, the onset of response to dupilumab for AA may be slower than that for AD, potentially requiring >3 months of administration to determine its efficacy. This finding is consistent with the results of a study by David et al. (9), in which children with AA who received dupilumab achieved a decrease in SALT scores as early as week 24. Finally, as relapse of AA after initial short-term treatment was observed in our case, maintenance therapy may be required after achieving early benefits to prevent recurrence after the discontinuation of drug administration.

The current study had some limitations, including the lack of detection of lymphocyte subsets and dominant cytokines in the scalp and/or blood circulation before and during the treatment period. Such data can be used to determine whether Th1 or Th2 skewing played a dominant role in this patient, thereby facilitating the correct selection of treatment regimen(s). Furthermore, because AA could also spontaneously resolve, given that our patient's condition has been under stable control for >1 year, the impact of extending the dupilumab treatment interval on the recurrence of AA still needs to be determined through a longer follow-up period.

## Conclusion

The findings of this case study highlighted the long-term efficacy and safety of dupilumab in a young child with severe ophiasis-pattern AA. However, given the paradoxical effects of dupilumab on AA treatment, possible predictors of clinical outcomes, including serum IgE levels and concomitant atopic diseases, should be considered on an individualized basis. The onset of the response to dupilumab in AA may be slower than that in AD. Maintenance therapy after the observation of early benefits may be required to prevent recurrence after discontinuation of drug administration.

## Patient perspective

As the mother of a 5-year-old boy with severe alopecia areata, I was understandably very concerned about his condition. He developed a serious inferiority complex and numerous hospital visits and topical medications for his alopecia areata offered no curative effects. Although the use of dupilumab for the treatment of alopecia areata in young children has yet to be fully established, I agreed with the doctor's plan regarding the use of this drug in the treatment of my son. After three months of treatment with dupilumab, there was no improvement in my son's alopecia areata. I was frustrated by this lack of progress and requested that the dupilumab treatment be discontinued. However, in the fourth month, I was pleasantly surprised to find a significant improvement in my son's condition with almost all of his scalp covered with new hair. I could not determine whether this improvement involved a natural remission of the alopecia areata or a delayed effect of the dupilumab. As this improvement proved to be temporary (lasting less than a month), this convinced me that it was the delayed therapeutic effect of dupilumab which was responsible for his improvement. More than half a year has passed since the resumption of his monthly dupilumab treatments and my son's alopecia areata has completely recovered. Moreover, he has also regained his confidence and emotional well-being. Now, after more than one year of treatment, I have not observed any adverse reactions in my son and I am very satisfied with this treatment method.

## Data Availability

The raw data supporting the conclusions of this article will be made available by the authors, without undue reservation.

## Supplementary material

Supplementary Figure S1 Dermoscopy revealed the presence of exclamation mark hairs, black dots, and broken hairs.
