# Supplementary figures and images for: Case Report: Severe ophiasis-pattern alopecia areata with concomitant atopic dermatitis in a 5-year-old boy successfully treated with dupilumab
Source: Front Pediatr. 2025 Feb 10;13:1517769. doi: 10.3389/fped.2025.1517769 (PMC11847702; doi:10.3389/fped.2025.1517769)

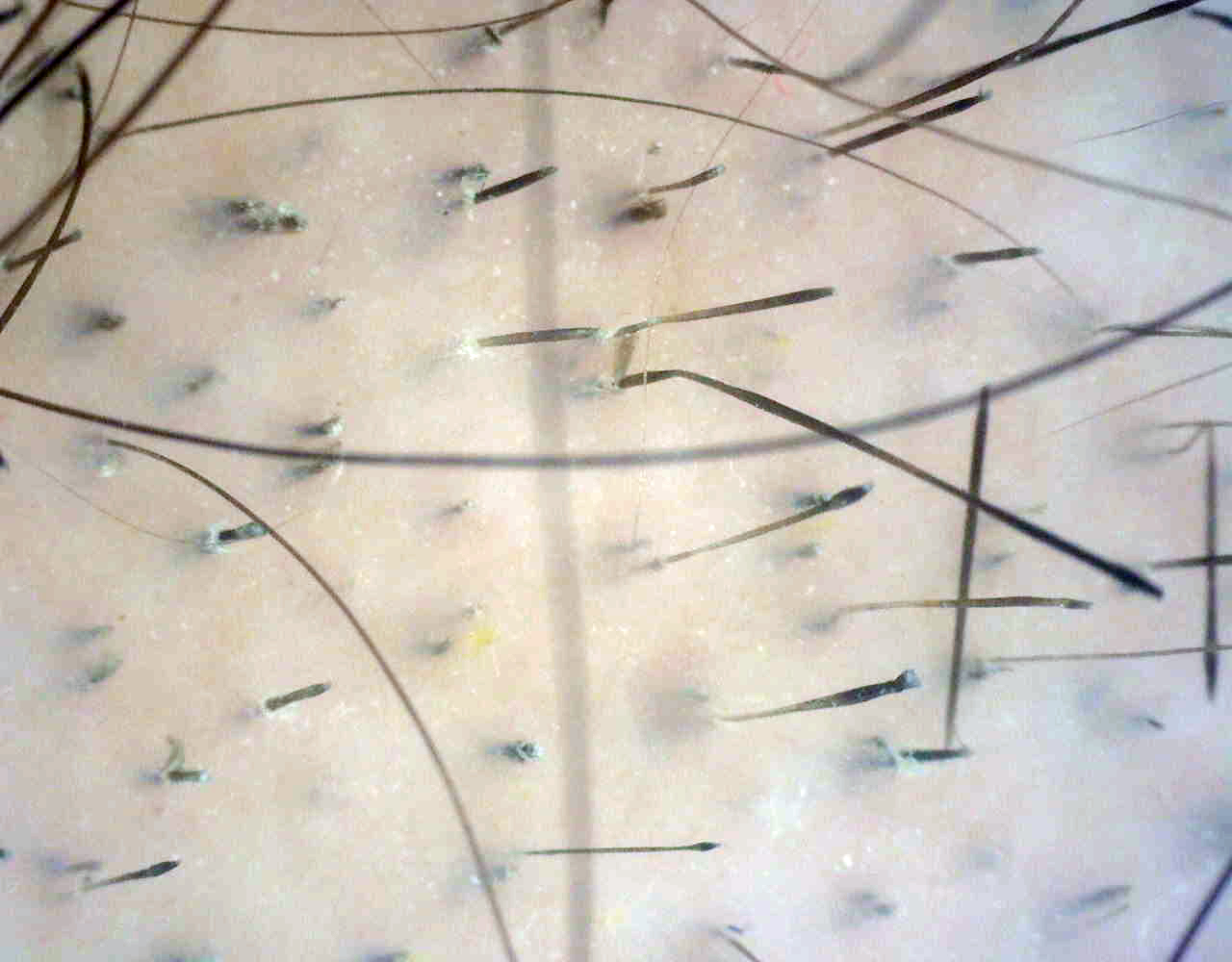

Supplement: Supplementary Figure S1 — Dermoscopy revealed the presence of exclamation mark hairs, black dots, and broken hairs. [file Image1.jpeg]
